# Spontaneous and evoked angiotensin II sniffer cell activity in the lamina terminalis in vitro

**DOI:** 10.1152/ajpregu.00227.2023

**Published:** 2024-08-12

**Authors:** George E. Farmer, J. Thomas Cunningham

**Affiliations:** Department of Physiology and Anatomy, University of North Texas Health Science Center at Fort Worth, Fort Worth, Texas, United States

**Keywords:** angtiotensin, circumventricular organ, hypertension, hypothalamus, peptide

## Abstract

Angiotensin II (ANG II) has been shown to have central nervous system effects. Although tissue renin-angiotensin systems (RAS) have been demonstrated in multiple tissues, the existence of a brain RAS is still a matter of debate. These studies test for angiotensin release from brain slices prepared from adult male Sprague-Dawley rats and male and female renin knock-out rats using Chinese hamster ovary cells modified to express both the angiotensin II type 1 receptor and a fluorescent calcium indicator. Sniffer cells were placed on the slices and calcium transients were measured from those located on or adjacent to the median preoptic nucleus with and without stimulation of the subfornical organ. Bath application of tetrodotoxin (1 µM) significantly attenuated spontaneous events while abolishing evoked sniffer cell activity. Bath application of dl-AP4 (10 µM, glutamatergic antagonist) did not affect either spontaneous or evoked release. Incubating the slices with fluorocitrate to inactive astrocytes did not influence sniffer cell activity in the MnPO. Pharmacological experiments indicate that ANG II release is largely both renin (aliskiren 10 µM) and ACE-1 (captopril 100 µM) dependent. However, experiments with brain slices prepared from male and female Renin knock-out rats suggest that alternative synthetic pathways may exist. Finally, these studies demonstrate that increases in ANG II release are observed following 7 days of chronic intermittent hypoxia. These studies suggest the existence of a tissue-specific RAS in the brain that involves canonical and alternative ANG II synthetic pathways and is upregulated in an animal model of sleep apnea.

**NEW & NOTEWORTHY** These studies used Chinese hamster ovary cells that were cloned to express an angiotensin receptor (*At1ra*) and a calcium indicator (R-GECO) to detect the release of angiotensin from brain slices containing the lamina terminalis of rats. Some of the experiments use tissue from renin knockout rats. The results support the existence of an angiotensin system in the brain that may involve alternative synthetic pathways and is upregulated by intermittent hypoxia.

## INTRODUCTION

The renin-angiotensin system (RAS) is one of the main hormone systems that regulates blood pressure and body fluid homeostasis (1–3). The canonical RAS uses renin as the rate-limiting step to cleave angiotensinogen into angiotensin I. Angiotensin-converting enzyme (ACE) changes angiotensin I to angiotensin II. Angiotensin II (ANG II) is the main circulating effector peptide for this system (2–4). Drugs that prevent the synthesis of ANG II or block angiotensin receptors are commonly prescribed for primary hypertension (1, 5). It has been established that, in addition to the canonical circulating RAS, several local tissue RASs have been described (4). Of these, the most controversial is probably the brain RAS (6–8). Several components of the canonical RAS are not abundant in the central nervous system (CNS), which has led several groups to propose that components of the peripheral RAS are transported into the CNS or that the brain uses alternative pathways to generate angiotensin peptides (7–9). Most of the machinery necessary for ANG II synthesis has been found in the brain. Angiotensinogen has been reported in subfornical organ (SFO) neurons and astrocytes (10–12). ACE has also been shown in brain tissue (12–14). However, while renin is not highly expressed in the CNS, the prorenin receptor has been proposed as an alternative pathway to convert angiotensinogen into angiotensin I (9, 15). In the CNS, additional neuroactive peptides are derived from ANG II by aminopeptidases (16, 17).

Although the existence of ANG II as a neurotransmitter is still a subject of debate, ANG II has been shown to influence blood pressure, hydromineral balance, memory, cognition, and kidney function. Circulating ANG II acts through the circumventricular organs of the brain such as the subfornical organ (SFO), organum vasculosum of the lamina terminalis (OVLT), and area postrema (AP) to modify brain functions (18). It has been proposed that centrally synthesized or transported ANG II acts as a neurotransmitter on the median preoptic nucleus (MnPO) and the paraventricular nucleus of the hypothalamus (7, 19, 20). Recently, it has been shown that ANG II may influence the uptake of glutamate by astrocytes and that pretreating rats with fluorocitrate reduces the effects of central ANG II injections on vasopressin release and salt appetite (21). This suggests that astrocytes contribute to ANG II signaling in the CNS. However, mechanisms controlling central ANG II signaling and how changes in control of central ANG II signaling can contribute to pathophysiology have not been determined.

A previous study demonstrated that Chinese Hamster Ovary cells can be genetically modified with angiotensin receptors and fluorescent calcium indicators and used to measure angiotensin peptide activity in brain slices in vitro (22). Using these ANG-sensitive sniffer cells, we showed central ANG II events in the MnPO that were activity dependent and blocked by tetrodotoxin (22). Based on our previous work, we tested whether ANG II is released in an activity-dependent manner that is codependent on glutamate (Glu) or astrocyte signaling. These studies also tested the hypotheses of whether or not central ANG II signaling is both renin and ACE1-dependent or if it might also rely on transport or alternative synthesis pathways in the absence of renin. Finally, this study tested whether or not 7 days of chronic intermittent hypoxia (CIH), an animal model of the hypoxemia related to obstructive sleep apnea, increases central ANG II release in the MnPO. CIH was used here because, in male rats, it produces a sustained increase in blood pressure that is dependent on central angiotensin receptors (23–25). Our previous studies have also shown that in male rats CIH increases the gene expression of *Ace1* in the MnPO, which could lead to an increase in ANG II synthesis (13, 14, 26).

## METHODS

### Animals

Experiments were performed according to the National Institutes of Health *Guide for the Care and Use of Laboratory Animals* (8th edition) under protocols approved by the University of North Texas Health Science Center Institutional Animal Care and Use Committee. Adult (6-wk-old, 250–300 g) male Sprague-Dawley rats (Charles River Laboratory, Wilmington, MA) were individually housed in temperature-controlled rooms (22–25°C) with a 12 h light-dark cycle with the light phase lasting from 0700 to 1900 h and ad libitum access to water and standard rat chow (LabDiet, St. Louis, MO). In addition, male and female renin knockout rats (SS.SS *Ren*1-m1, age 2–4 mo) were obtained from the Medical College of Wisconsin Rat Research Model Service Center (Milwaukee, WI). After shipping, SS.SS Ren1-m1 rats were given 2 wk to acclimate to the vivarium before experiments. SS.SS *Ren*1-m1 were group housed and given ad libitum access to water and standard rat chow. All rats were housed in plastic housing cages connected to a closed-air filtration system. Cages were filled with corn cob bedding and shredded paper for enrichment and were changed weekly. All surgeries were performed using aseptic technique, and a nonsteroidal anti-inflammatory drug carprofen (Bio-Serv, Flemington, NJ, 2 mg tablet orally) was given before and after surgery for pain management. At the time of euthanasia, vaginal smears were collected from female SS.SS *Ren*1-m1 rats to determine their stage of the estrous cycle by cytological evaluation as previously described (27).

### Microinjections

**Figure 1.**
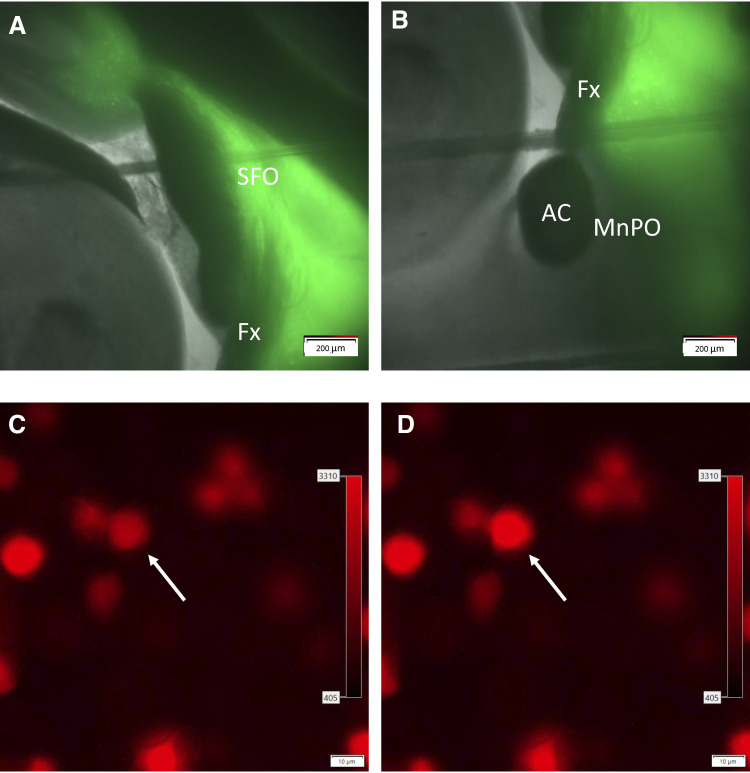
Expression of channel rhodopsin in the SFO (*A*) projections to the MnPO (*B*). Sniffer cells placed on the MnPO (*C*) show an increase in fluorescence in the presence of ANG II (*D*). AC, anterior commissure; ANG II, angiotensin II; Fx, fornix; MnPO, median preoptic nucleus; SFO, subfornical organ.

The channel rhodopsin expressing viral vector [AAV2-hSyn-ChR2(E123A)-eYFP-WPRE] used in these experiments was obtained from the UNC Vector Core as provided by the Deisseroth Laboratory (Fig. 1, *A* and *B*). The virus was injected undiluted at a titer of 1.1 × 10^12^ genomic particles/mL. Sprague-Dawley rats were anesthetized with 2% isoflurane (mixed with 95% O_2_-5% CO_2_), and their scalps were shaved and disinfected with alcohol and betadine. Each rat was placed in a Kopf (Tujunga, CA) stereotaxic head frame. To ensure accurate injections, skulls were leveled between bregma and lambda (28). The coordinates used for the SFO injections were 1.5 mm posterior, 0.0 mm lateral, and 5.5 mm ventral from bregma (24). After a burr hole was drilled at the site of injection, a 30-gauge steel injector was lowered to the SFO and 200–300 nL of AAV was delivered at a rate of 200 nL/min. The injector was connected to a Hamilton 5 µL syringe (No. 84851 Hamilton, Reno, NV) by calibrated polyethylene tubing that was used to determine the injection volume. The injector remained inserted for 5 min before being slowly withdrawn. Gel foam was packed into the opening in the cranium and absorbable antibiotic sutures were used to close the incision.

### Chronic Intermittent Hypoxia

Male Sprague-Dawley rats were exposed to 7 days of chronic intermittent hypoxia (CIH) as previously described (24, 29). All rats were transferred to the room containing the CIH chambers for at least one week after arriving in the vivarium. Rats exposed to CIH were housed in an 8 in. × 9 in. cage that was placed inside of custom Plexiglas chambers. Rats were housed in these chambers for a 5-day baseline period before the start of the CIH protocol. The CIH protocol consisted of 6-min cycles; 3 min of 21% oxygen room air pumped in, 90 s of nitrogen pumped in to lower the chamber O_2_ to 10% oxygen, and then 90 s of maintenance at 10% oxygen. This cycle repeated 10 times per hour, 8 h a day (0800–1600 h) for 7 consecutive days. Animals were exposed to room air for the remainder of the day. Normoxic controls were housed in the same room under similar conditions but were only exposed to room air.

### Brain Slice and Sniffer Cell Preparation

Sprague-Dawley and SS.SS Ren1-m1 rats were anesthetized with 2% isoflurane (mixed with 95% O_2_-5% CO_2_) and decapitated two to three weeks following microinjections. Sagittal slices (300 µm) containing the MnPO and the SFO were cut using a Microslicer DTK Zero 1 (Ted Pella, Inc., Redding, CA) in ice-cold (0–1°C), oxygenated (95% O_2_-5% CO_2_) cutting solution consisting of (in mM): 3.0 KCl, 1.0 MgCl_2_·6H_2_O, 2.0 CaCl_2_, 2.0 MgSO_4_, 1.25 NaH_2_PO_4_, 26 NaHCO_3_, 10 d-glucose, and 206 sucrose (300 mosmol, pH 7.4). Slices were incubated at room temperature in oxygenated (95% O_2_-5% CO_2_) artificial cerebrospinal fluid (aCSF) containing (in mM): 126 NaCl, 3.0 KCl, 2.0 CaCl_2_, 2.0 MgSO_4_, 1.25 NaH_2_PO_4_, 26 NaHCO_3_, 10 and d-glucose (300 mosmol, pH 7.4) for a minimum of 1 h before recording. During this 1-h incubation period, ANG sniffer cells that were previously grown to 80% to 90% confluency on 60-mm or 100-mm plates were trypsinized, suspended in 1× PBS, and counted using a hemocytometer. After being spun down (1,600 rpm), ANG sniffer cells were suspended in aCSF at a concentration of 1.3–2.5 × 10^6^ cells/mL as previously described (22).

The ANG sniffer cells used in these experiments were from a stable cell line created by transfecting genetically female Chinese Hamster Ovary cells (ATTCC, Manassas, VA) with commercially available plasmids for the angiotensin type 1 a receptor (plasmid Agtr1a/CMV6-Entry Cat no. RR215252 from OriGene, Rockville, MD) and R-GECO (CMV-NLS-R-GECO from Robert Campbell, University of Alberta, plasmid no. 32462, Addgene) that were prepared as previously described (22).

Sagittal slices containing the MnPO and SFO were transferred to a submersion recording chamber and superfused with aCSF (1–2 mL/min, 31 ± 1°C) (30). Slices were visualized using an upright microscope (BX50WI, Olympus) equipped for epifluorescence and differential interference contrast optics. During a temporary interruption of aCSF flow slices were seeded with a 300-µL suspension of sniffer cells. Sniffer cells were allowed 3 min to settle before aCSF flow was resumed. A concentric platinum-iridium bipolar stimulating electrode (no. CBBRE75, FHC, Bowdoin, ME) was placed in the fiber path between the SFO and the MnPO in slices from uninjected rats or from rats where the channel rhodopsin injection missed the SFO. Slices were electrically stimulated at 100 Hz for 1 s (0.1-ms pulse duration). In slices from rats with successful channel rhodopsin injections, borosilicate glass micropipettes (1–3 µm tip) containing aCSF as the internal solution and a fiber optic cable in the tip (0.5 mm, A-M Systems) of the micropipette were positioned at the MnPO. SFO terminals were activated using a 530 nm laser (PSU-III-LED, Laserglow Technologies) using the following parameters: 20 Hz, 1 s, 2 ms pulse, 5 mW. Fluorescent images of sniffer cells positioned over the MnPO were imaged using CellSens Dimensions software (Olympus). Images were captured at 1-s intervals and measurements of fluorescent intensity were taken (Fig. 1, *C* and *D*).

### Drugs

The compounds fluorocitrate, aliskiren, and captopril were purchased from Sigma-Aldrich (St. Louis, MO). The compounds tetrodotoxin (TTX) and dl-AP4 (dl-2-amino-4-phosphonobutyric acid), were purchased from Tocris (Minneapolis, MN). Stock solutions of aliskiren (10 µM) were prepared in methanol while stock solutions of captopril (100 µM), TTX (1 µM), and dl-AP4 (10 µM) were prepared in aCSF. All stock solutions were diluted in aCSF to working concentrations.

### Data Analysis

The frequency of spontaneous changes in the fluorescence intensity of identified regions of interest located above the MnPO were analyzed in normal aCSF and the presence of different drugs. Changes in the frequency of sniffer cell calcium transients were also measured following electrical or optogenetic stimulation of the subfornical organ. In addition, the analysis of evoked sniffer cell responses consisted of amplitude and area under the curve measurements.

Data were analyzed for group or group and drug-dependent effects using a Kruskal–Wallis one-way ANOVA on ranks followed by Dunn’s post hoc tests or two-way ANOVA followed by Student–Newman–Keuls (SNK) post hoc tests based on whether the data passed tests for normality or variability. Analyses were performed using SigmaPlot software (v 12.0, Systat Software, Inc., San Jose, CA). Data are reported as the means ± 1 SE.

## RESULTS

### SFO Evoked Responses

**Figure 2.**
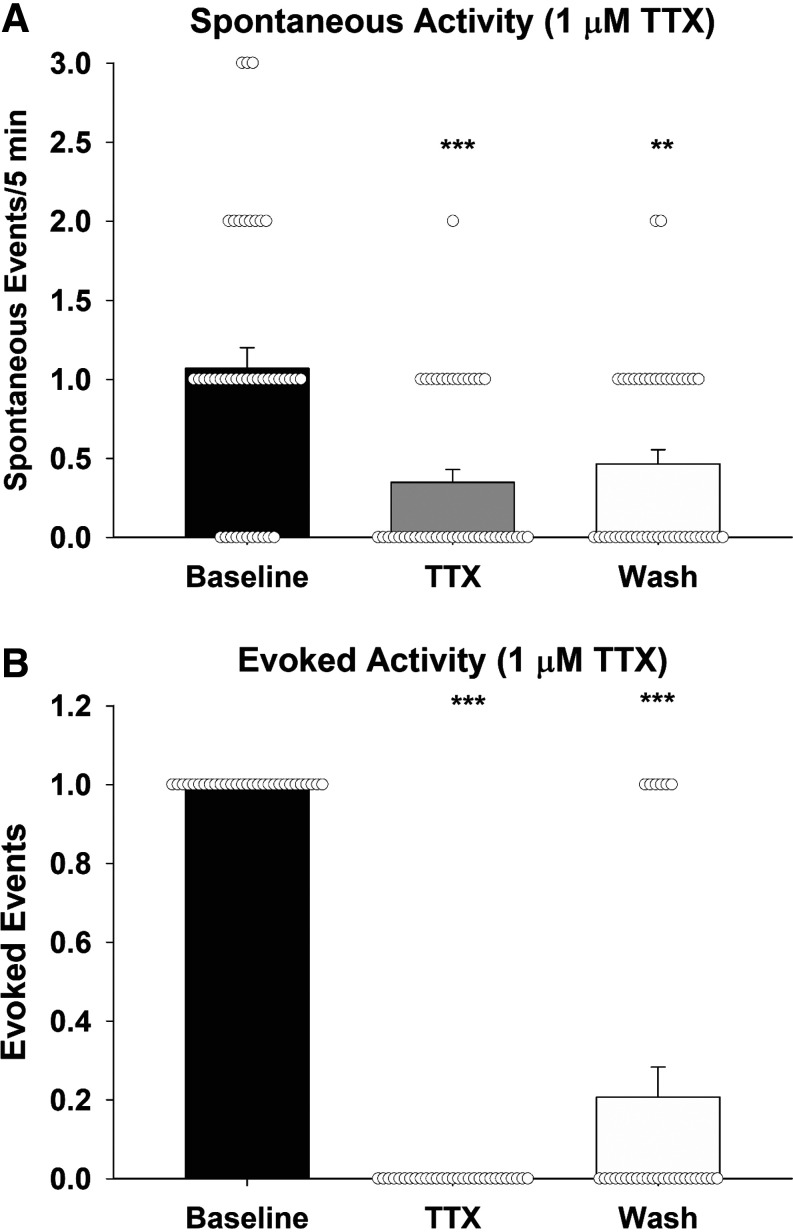
Release of ANG II is activity dependent. Spontaneous sniffer cell responses are reduced in the presence of 1 µM TTX (*A*). TTX completely blocks evoked sniffer cell responses (*B*). ***P < 0.001, **P < 0.01 compared with baseline. ANG II, angiotensin II; TTX, tetrodotoxin.

A previous study has shown that bath application of TTX attenuates spontaneous calcium transients in sniffer cells placed on the MnPO (22). This study tests the ability of TTX to block sniffer cell responses in response to optogenetic stimulation of the SFO-MnPO pathway. Sniffer cells were recorded from sagittal MnPO slices (8 slices from 6 rats). Twenty sniffer cells showed spontaneous activity (1.1 spikes/5 min) during baseline recordings which was reduced to 0.35 spikes/5 min during bath application of 1 µM TTX (Fig. 2*A*). Spontaneous activity returned to 0.47 spikes/5 min following 5 min washout of TTX [Kruskal–Wallis one-way ANOVA on ranks; H(2) = 21.36, *P* < 0.001]. Furthermore, 30 sniffer cells showed evoked responses following optogenetic stimulation of the SFO, and bath application of TTX completely blocked the evoked sniffer cell responses (Fig. 2*B*). Evoked sniffer cell responses returned in 20% of the recordings following washout of the TTX [Kruskal–Wallis one-way ANOVA on ranks; H(2) = 66.44, *P* < 0.001].

**Figure 3.**
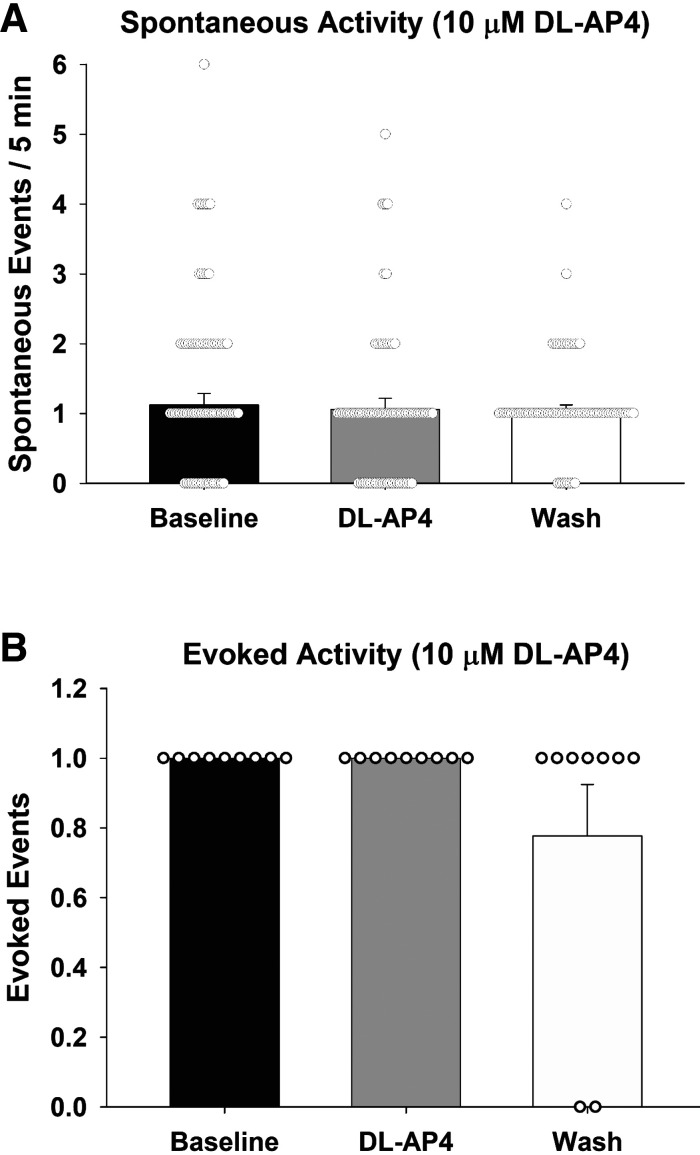
Both spontaneous (*A*) and evoked (*B*) sniffer cell responses are not influenced by broad-spectrum blockade of glutamatergic signaling. dl-AP4, dl-2-amino-4-phosphonobutyric acid.

To further investigate the release of ANG II in the MnPO, tests addressed the contribution of glutamate receptors in sniffer cell responses evoked by optogenetic stimulation of the SFO. A total of 78 spontaneously active sniffer cells were recorded in 7 slices from 6 rats (Fig. 3*A*). Spontaneous activity during the 5-min baseline recording period averaged 1.12 spikes (±0.17 spikes). After bath application of dl-AP4, sniffer cells exhibited a mean of 1.06 (±0.16) spikes during the 5-min recording. After the 5-min washout of DL-AP4, sniffer cells showed a mean of 1.00 (±0.12) spontaneous responses. There was no significant effect of dl-AP4 on the spontaneous activity of sniffer cells in the MnPO [Kruskal–Wallis one-way ANOVA on ranks; H(2) = 0.12, *P* = 0.94].

Furthermore, nine sniffer cells showed evoked responses following optogenetic stimulation of the SFO (Fig. 3*B*). However, the bath application of dl-AP4 did not block optogenetic-evoked sniffer cell responses [Kruskal–Wallis one-way ANOVA on ranks; H(2) = 4.16, *P* = 0.13].

### Fluorocitrate

**Figure 4.**
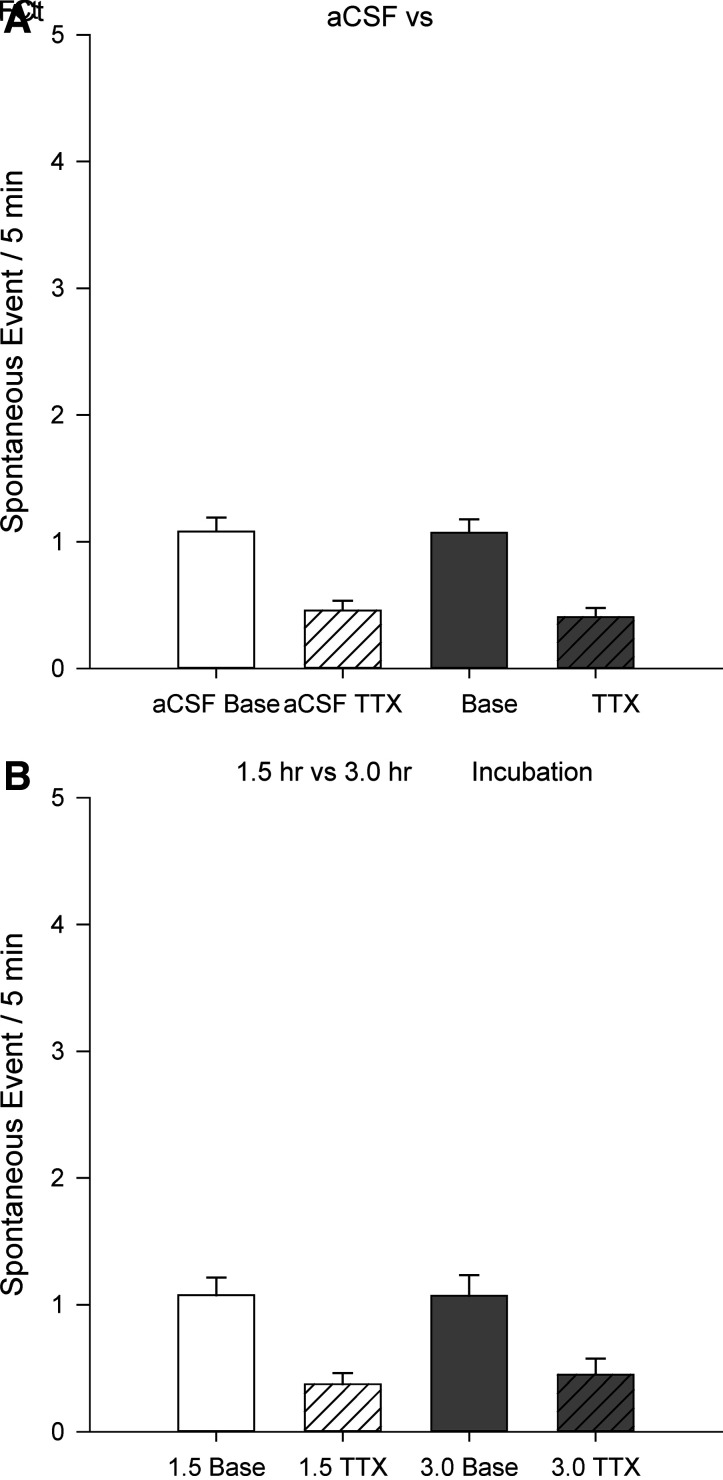
Inactivation of astrocytes does not influence baseline spontaneous sniffer cell activity or TTX-mediated reductions in sniffer cell activity (*A*). There are no time-dependent effects of astrocyte inactivation on spontaneous sniffer cell activity (*B*). aCSF, artificial cerebrospinal fluid; FCt, fluorocitrate; TTX, tetrodotoxin.

This study further investigated the potential contribution of astrocytes in ANG II signaling in the MnPO. In control slices (Fig. 4*A*), sniffer cells (*n* = 61) showed baseline spontaneous activity of 1.08 ± 0.11 spikes/5 min that was reduced to 0.46 ± 0.08 spikes/5 min following bath application of 1 µM TTX. Sniffer cells (*n* = 68) on slices incubated in 100 µM fluorocitrate (FCt) showed baseline spontaneous activity of 1.07 ± 0.11 spikes/5 min that was reduced to 0.41 ± 0.07 spikes/5 min following bath application of 1 µM TTX. Similar to above, there was a significant effect of TTX application [two-way ANOVA *F*(1,256) = 48.19, *P* < 0.001] but no effect of FCt incubation [two-way ANOVA *F*(1,256) = 0.11, *P* = 0.74]. Furthermore, tests examined the possible time-dependent effects of FCt incubation (Fig. 4*B*). Slices incubated for 1.5 h with FCt before sniffer cell recordings had baseline spontaneous sniffer cell (*n* = 39) activity of 1.08 ± 0.14 spikes/5 min that was reduced to 0.38 ± 0.09 spikes/5 min by bath application of 1 µM TTX. Slices incubated for 3 h with FCt before sniffer cell recordings had baseline spontaneous sniffer cell (*n* = 29) activity of 1.07 ± 0.16 spikes/5 min that was reduced to 0.45 ± 0.13 spikes/5 min followed by 1 µM TTX. The analysis of these data indicated a significant effect of TTX application [two-way ANOVA *F*(1,141) = 27.23, *P* < 0.001] but not FCt incubation [two-way ANOVA *F*(1,141) = 0.06, *P* = 0.80].

### Renin-Angiotensin System

**Figure 5.**
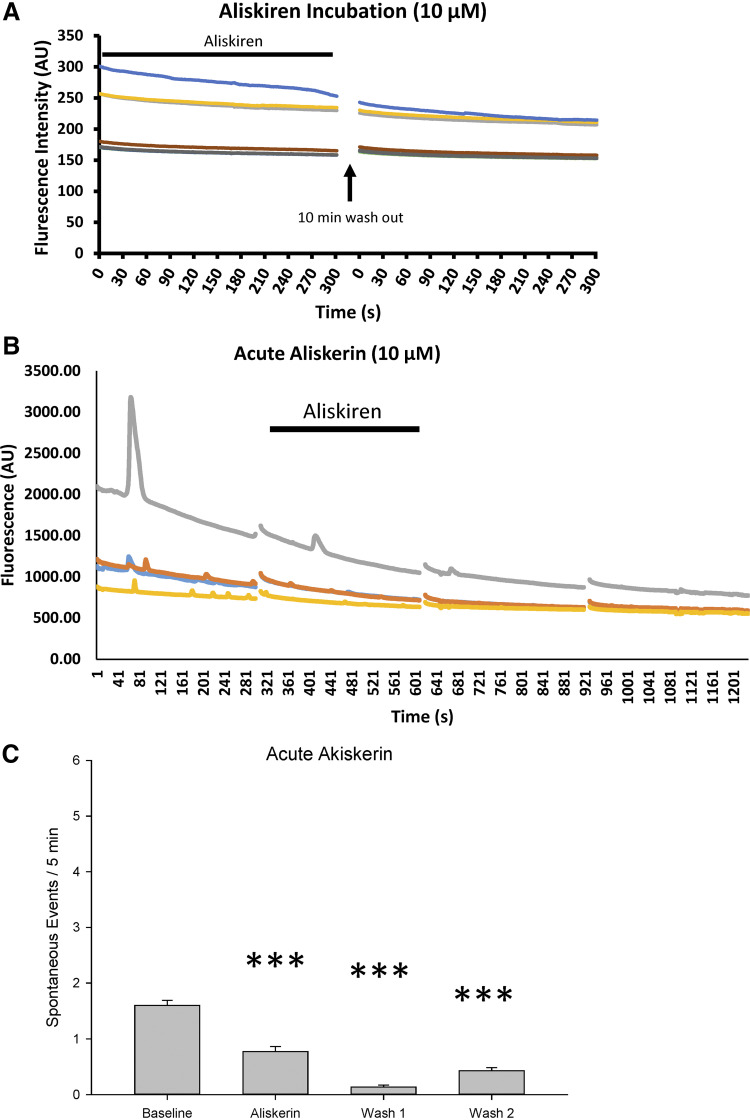
Renin inhibition reduces spontaneous sniffer cell activity. Prolonged exposure to aliskerin completely abolishes spontaneous sniffer cell activity in MnPO slices (*A*). Acute application of aliskerin produces a delayed, but marked, decrease in spontaneous sniffer cell activity (*B*). A summary of the time-dependent reduction in sniffer cell activity is presented in *C*. ***P < 0.001 compared with baseline. MnPO, median preoptic nucleus.

To test the role of renin in sniffer cell activity, brain slices from two rats were incubated in 10 µM aliskiren for at least 1 h before application and recording of sniffer cells after which the slices were maintained in aCSF containing 10 µM aliskiren during sniffer cell recordings. At no time were spontaneous or evoked sniffer cell responses observed during this prolonged aliskiren exposure (Fig. 5*A*). Because of the failure to observe sniffer cell responses during the chronic exposure of aliskiren, a more acute aliskiren protocol was adopted that was similar to the TTX and dl-AP4 protocols.

Spontaneous responses were recorded from sniffer cells (*n* = 119) on the MnPO in five slices from five rats (Fig. 5*B*). There was a significant effect of aliskerin treatment on the spontaneous activity of sniffer cells [Kruskal–Wallis one-way ANOVA on ranks; H(3) = 187.54, *P* < 0.001]. During the 5-min baseline recording (Fig. 5*C*), spontaneous sniffer cell activity was 1.60 ± 0.09 spikes. The spontaneous activity was reduced slightly following wash-in of 10 µM aliskiren to 0.77 ± 0.09 spikes (*P* < 0.001; Fig. 5*C*). Interestingly, spontaneous sniffer cell activity was still significantly reduced during the 5 min washout period (0.13 ± 0.03 spikes; *wash 1*, *P* < 0.001; Fig. 5*C*). Activity was still significantly reduced 15 min following washout of aliskerin (0.43 ± 0.06 spikes, *wash 2*, *P* < 0.001; Fig. 5*C*). However, sniffer cell activity started to return toward baseline levels following 15 min of aliskerin washout (*wash 1* vs. *wash 2*, *P* < 0.05).

**Figure 6.**
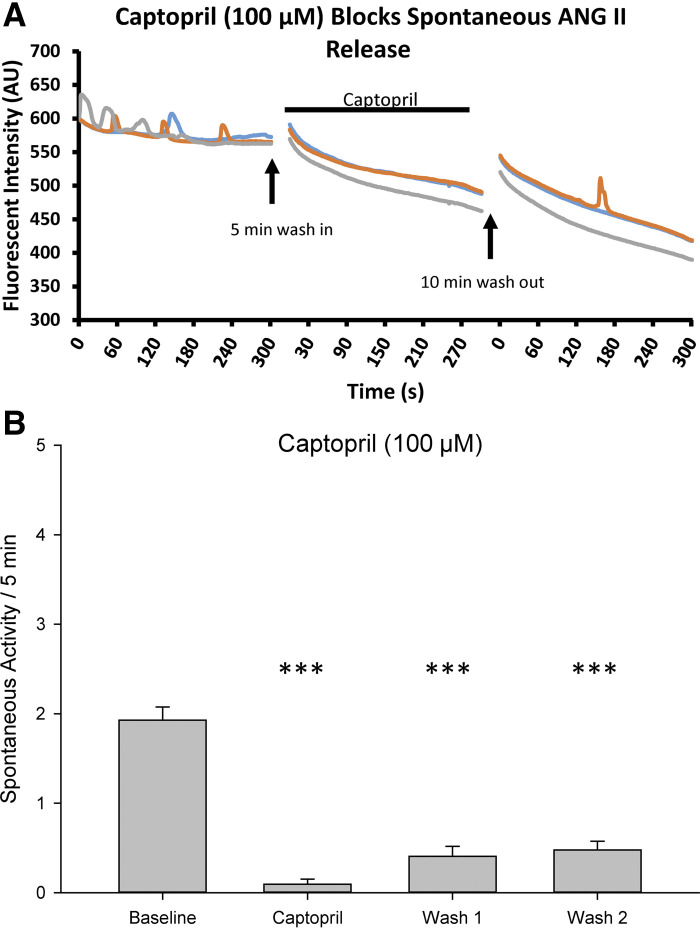
Inhibition of ACE1 inhibits spontaneous sniffer cell activity. Bath application of captopril reduces the spontaneous sniffer cell activity (*A*). Spontaneous sniffer cell activity recovers following washout of captopril (*B*). ACE1, angiotensin-converting enzyme-1; ANG, angiotenisin II. ***P < 0.001 compared with baseline.

The contribution of angiotensin-converting enzyme in sniffer cell activity was also tested. Spontaneous sniffer cell responses were recorded from the MnPO in seven slices from five rats (Fig. 6*A*). Baseline recording of spontaneous sniffer cell (*n* = 42) activity was 1.93 ± 0.15 spikes which was significantly reduced to 0.09 ± 0.06 spikes following bath application of 100 µM captopril [Kruskal–Wallis one-way ANOVA on ranks; H(3) = 91.36, *P* < 0.001]. Spontaneous sniffer cell activity recovered following the 5-min washout period to 0.40 ± 0.11 spikes. After an additional 15-min washout, spontaneous sniffer cell activity further recovered to 0.48 ± 0.10 spikes. The increases in sniffer cell activity at 5 min (*wash 1*, *P* < 0.001) and 15 min following washout of captopril (*wash 2*, *P* < 0.001) were significant when compared with the baseline recording (Fig. 6*B*).

To further characterize the role of the brain RAS, MnPO slices were prepared from male and female Ren1-m1 rats. Experiments were performed using three different genotypes: wild-type (WT), heterozygous renin knockouts (Ren^+/−^), and homozygous renin knockouts (Ren^−/−^) (Table 1). In addition, female rats were tested to determine the stage of the estrous cycle before slice preparation (Table 2).

**Table 1.** Sex and genetic phenotypes of renin knockout rats used for these studies

Rat	Male	Female	Genotype Total
Wild type	4	4	8
Ren^+/−^	4	3	7
Ren^−/−^	2	5	7
Total	10	12	22

**Table 2.** Reproductive status and genotypes of female rats

Rat	Metestrus	Diestrus	Proestrus	Estrous
Wild type	2	0	1	1
Ren^+/−^	0	1	2	0
Ren^−/−^	1	2	2	0
Total	3	3	5	1

**Figure 7.**
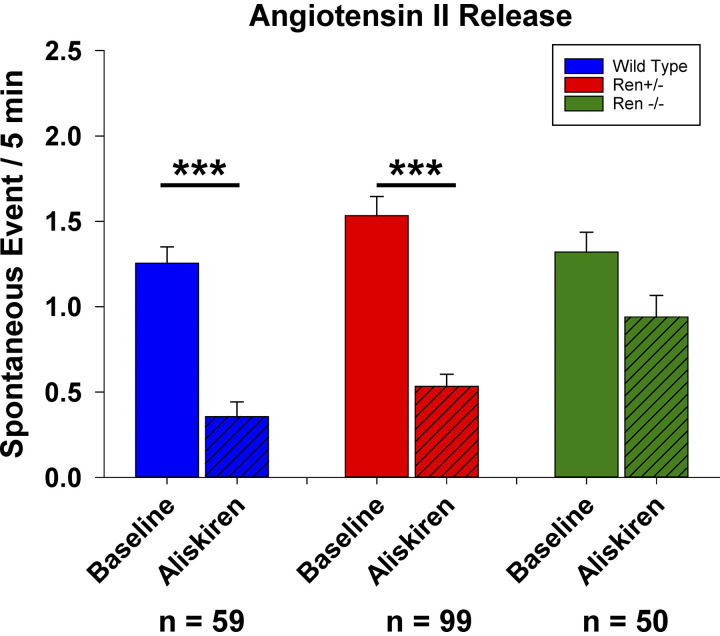
Renin inhibition reduces spontaneous sniffer cell activity in wild-type and heterozygous renin knockout rats but does not reduce spontaneous sniffer cell activity in homozygous renin knockout rats. ***P < 0.001 compared with baseline.

The spontaneous sniffer cell activity associated with the MnPO in slices prepared from WT Ren1-m1 rats (1.25 ± 0.10 spikes, *n* = 81) was comparable to the baseline activity of Sprague-Dawley rats described above (Fig. 7). In the WT rats, aliskiren reduced the spontaneous activity of sniffer cells (0.36 ± 0.08 spikes, *P* < 0.001). In experiments with slices prepared from Ren^+/−^ rats, baseline spontaneous sniffer cell activity (1.53 ± 0.11 spikes, *n* = 96) was similar to that of WT rats, and spontaneous activity was significantly attenuated following acute application of aliskiren (0.53 ± 0.07 spikes, *P* < 0.001). In experiments with MnPO slices from *Ren*^−/−^ rats, baseline sniffer cell activity was not different (1.32 ± 0.12 spikes, *n* = 50) as compared with data from experiments using slices from WT or *Ren*^+/−^ rats but acute aliskiren application did not significantly affect spontaneous sniffer cell activity (1.04 ± 0.12 spikes, *P* = 0.06).

**Figure 8.**
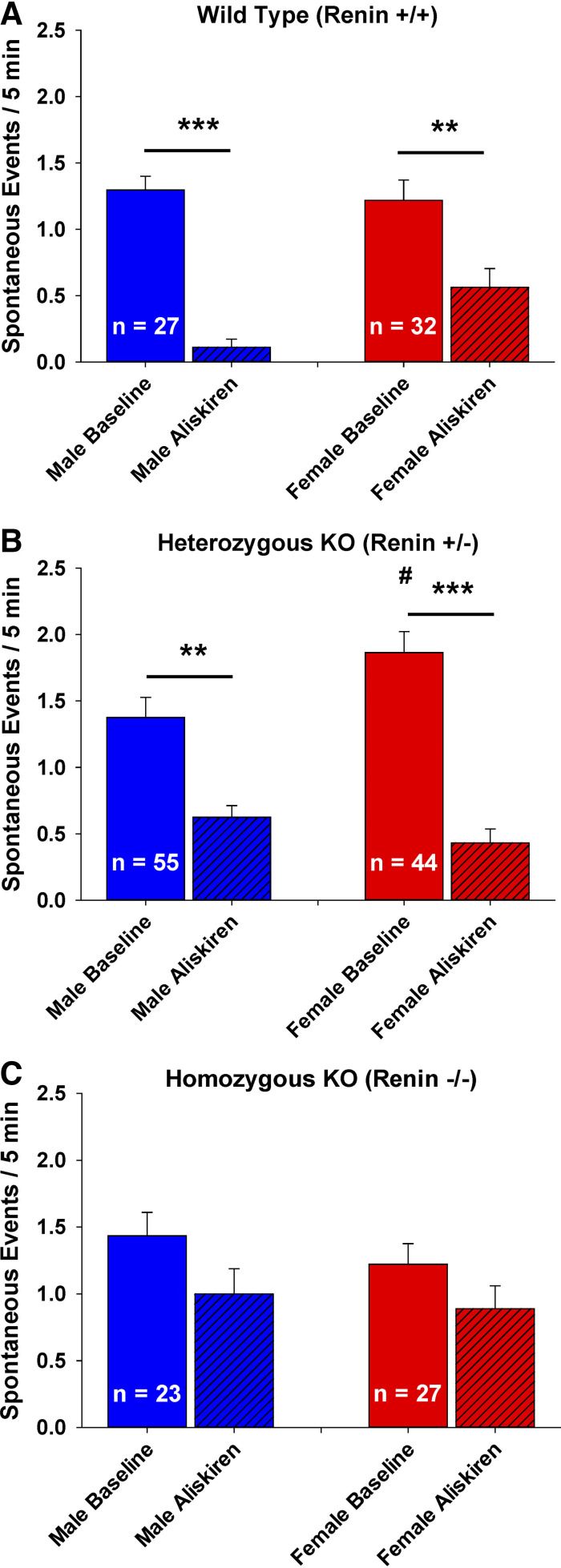
Sex differences in the role of renin on spontaneous sniffer cell activity are observed in MnPO slices. Males exhibit a more pronounced decrease in sniffer cell activity following renin inhibition than females in wild-type rats (*A*). In heterozygous renin KO rats, females exhibit a greater basal sniffer cell activity than males but both males and females show similar sniffer cell activity in response to renin inhibition (*B*). Male and female homozygous renin KO rats show similar basal levels of sniffer cell activity that is insensitive to renin inhibition (*C*). ***P < 0.001, **P < 0.01 compared with baseline. #*P* < 0.05 compared with the baseline from males. KO, knockout rats; MnPO, median preoptic nucleus.

There was a significant difference in spontaneous sniffer cell activity between WT groups when the male and female data was separated [Fig. 8*A*, Kruskal–Wallis one-way ANOVA on ranks; H(3) = 51.82, *P* < 0.001]. Sniffer cells from WT rats showed a significant aliskiren-mediated reduction in spontaneous activity in both the males (*n* = 27, *P* < 0.001) and females (*n* = 32, *P* < 0.01). Furthermore, data showed that there was no difference in baseline spontaneous sniffer cell activity between male and female WT rats (*P* = 1.00). In addition, there was no difference in aliskiren-mediated inhibition of sniffer cell activity between male and female rats (*P* = 0.19). There was also a significant difference in spontaneous sniffer cell activity between *Ren*^+/−^ groups (Fig. 8*B*) when the male and female data were separated [Kruskal–Wallis one-way ANOVA on ranks; H(3) = 61.44, *P* < 0.001]. Sniffer cells from female Ren^+/−^ rats (*n* = 44) showed an elevated baseline spontaneous sniffer cell activity compared with the baseline activity in sniffer cells from female WT rats (*P* < 0.001). However, there was no difference in baseline sniffer cell activity between male *Ren*^+/−^ (*n* = 55) and WT (*n* = 27) rats, nor was there a difference between male and female *Ren*^+/−^ rats. Aliskiren reduced the spontaneous activity of sniffer cells in both the male (*P* < 0.01) and female (*P* < 0.001) Ren^+/−^ rats but the aliskiren-mediated reductions in the spontaneous activity were not different between male and female *Ren*^+/−^ rats (*P* = 0.98). There was no significant difference in spontaneous sniffer cell activity between *Ren*^−/−^ groups (Fig. 8*C*) when the male and female data was separated [Kruskal–Wallis one-way ANOVA on ranks; H(3) = 6.69, *P* = 0.08]. Basal spontaneous sniffer cell activity was similar between male and female rats and aliskiren did not significantly alter the spontaneous sniffer cell activity in both the male and female rats.

### Chronic Intermittent Hypoxia

**Figure 9.**
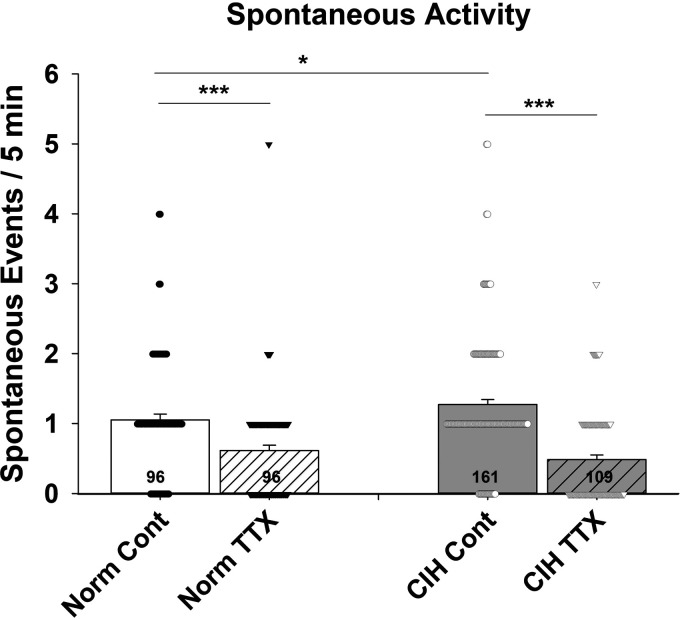
Chronic intermittent hypoxia increases basal spontaneous sniffer cell activity in the MnPO. The increase in basal sniffer cell activity following CIH is due to an increase in activity-dependent release of ANG II. ***P < 0.001, *P < 0.05. ANG II, angiotensin II; CIH, chronic intermittent hypoxia; MnPO, median preoptic nucleus; TTX, tetrodotoxin.

Angiotensin II release was assessed in the MnPO of male Sprague-Dawley rats following 7 days of CIH treatment (Fig. 9). Spontaneous sniffer cell activity was recorded from 10 MnPO slices in 8 CIH-treated rats and from 7 slices in 5 normoxia-treated rats. Baseline sniffer cell activity in MnPO slices from normoxia treated was 1.05 spikes/5 min (*n* = 96) and was reduced to 0.62 spikes (*n* = 96) in the presence of 1 µM TTX. In MnPO slices from CIH-treated rats, baseline spontaneous sniffer cell activity was 1.27 spikes/5 min (*n* = 161). Bath application of TTX reduced spontaneous sniffer cell activity to 0.49 spikes/5 min in MnPO slices from CIH-treated rats. There was a significant effect of treatment on spontaneous sniffer cell activity [one-way ANOVA; *F*(3, 458) = 26.85, *P* < 0.001]. Baseline spontaneous sniffer cell activity was increased following CIH when compared with normoxic controls (*P* < 0.05). Bath application of TTX attenuated spontaneous sniffer cell activity in the MnPO of both normoxic (*P* < 0.001) and CIH (*P* < 0.001)-treated rats to comparable levels. No differences were detected between normoxic and CIH-treated rats in evoked sniffer cell activity in MnPO.

## DISCUSSION

Spontaneous ANG II activity as assayed by ANG-sensitive sniffer cells in the MnPO has previously been shown to be, in part, activity-dependent and is reduced by bath application of TTX (22). Furthermore, ANG II release was detected by sniffer cells on the MnPO in response to optogenetic stimulation of the SFO. This suggests that a source of ANG II is from the SFO. However, the temporal resolution of the sniffer cell responses demonstrated here cannot determine if the ANG II release in response to SFO stimulation is due to synaptic release of ANG II from SFO terminals. It is possible that ANG II release in the MnPO is secondary to SFO-dependent activation and that ANG II may be released locally in response to SFO stimulation of the MnPO. To address this issue, a broad-spectrum glutamate receptor antagonist was bath applied to see if it would block the optogenetically induced sniffer cell responses. In this study, broad-spectrum glutamate block did not influence the spontaneous or evoked sniffer cell activity. Incubating the slices FCt, which is reported to metabolically inhibit astrocytes (31), did not significantly affect the activity of ANG II sniffer cells associated with the MnPO. These results indicate that the activation of the ANG II sniffer cells on the MnPO associated with SFO stimulation is not glutamate or astrocyte dependent.

In addition to addressing the source of ANG II in the MnPO, these studies investigated the synthetic pathways that are involved in the synthesis of brain derived ANG II. In Sprague-Dawley rats, acute application of the ACE1 inhibitor captopril was effective in reducing the activity of sniffer cells. Upon washout of captopril, sniffer cells exhibit a return toward baseline activity. Application of aliskiren was also sufficient to block spontaneous ANG II detection using sniffer cells. Interestingly, prolonged aliskerin incubation (>1 h) blocked nearly all spontaneous sniffer cell activity, which was unable to be recovered following washout of aliskerin. However, acute application of aliskerin was able to reduce the activity of the sniffer cells. In addition, the acute aliskerin-dependent inhibition of sniffer cell activity was delayed and peaked during the washout period following bath application of aliskerin. The delayed inhibitory effects of aliskerin combined with the more rapid inhibitory effects of captopril hint at the possibility for a compartmentalized synthesis of ANG II in the brain (Figs. 5 and 6). It is possible that ANG II precursors are synthesized in one cell type, for instance, astrocytes, which must then be transported to neuronal cells for further syntheses into ANG II. However, incubating brain slices in FCt had no effect on spontaneous sniffer cell activity or on the activity dependence of sniffer cell activity. The disparity in the inhibitory time course of ACE1 and renin inhibitors is yet to be determined but may be a function of enzyme turnover or of compartmentalized synthesis of ANG II within the neuron. Furthermore, although both captopril and aliskerin were effective in reducing sniffer cell activity, there was not a complete block of activity leaving the possibility of a minor role for alternative synthetic pathways. These aspects of the results are consistent with alternative pathways for angiotensin synthesis such as cathepins, chymase, and the prorenin receptor (15–17, 32).

Although the studies in untreated Sprague-Dawley rats suggest that canonical ANG II synthetic pathways may be used in the brain and that alternative synthetic pathways are not involved or minimally involved in the production of the central ANG II in this experimental setup it may be possible that alternate synthetic pathways are induced during various stresses or diseases such as salt loading, DOCA salt, or chronic intermittent hypoxia. To test the dependence of brain ANG II in the MnPO on canonical pathways brain slices were prepared from heterozygous and homozygous renin knock out rats. Observations show that spontaneous sniffer cell activity in the MnPO was dependent on renin activity in WT and *Ren*^+/−^ rats. Sniffer cell activity in the MnPO of *Ren*^−/−^ rats persisted and was comparable to activity observed in WT and *Ren*^+/−^. In addition, bath application of aliskerin did not reduce the activity of sniffer cells experiments with brain slices from the *Ren*^−/−^ rats.

Based on the experiments with the *Ren* KO rats, it is possible that there could be sex-based differences in the role of renin in central angiotensin sniffer cell activity in the MnPO. Although we observed statistically significant differences in the data suggesting ovarian hormones may influence renin-dependent ANG II synthesis and not renin-independent ANG II synthesis we acknowledge our study is limited by the number of female rats that were used and that the estrous cycle stage was only assessed at the time of sacrifice. These possible sex-based differences could warrant further investigation.

The lamina terminalis, which includes the SFO and MnPO, is involved in the sustained hypertension associated with CIH which can be blocked with electrolytic lesions of this region (26). Furthermore, central ANG II signaling in the lamina terminalis has been shown to contribute to sustained hypertension in CIH. Chronic intermittent hypoxia not only increases blood pressure but also the expression of FosB/ΔFosB in the lamina terminalis (29) in rats which can be blocked by central administration of the angiotensin II type 1 receptor (AT1aR) antagonist losartan (23). CIH increases circulating ANG II (33) and activation of ANG II receptors in the SFO, which are sensitive to circulating ANG II, contribute to CIH hypertension (24). In addition, blockade of AT1aR activation in the SFO blocks increases in MnPO ΔFosB associated with CIH. Increases in MnPO FosB are associated with increases in *At1ars* in the MnPO (34, 35) and blockade of MnPO FosB prevents increases in *At1ar* expression and hypertension associated with CIH (25). Similarly, blockade of AT1aR expression in the MnPO blocks CIH-dependent increases in FosB (25). The cyclical nature of AT1aR activation facilitating increased *At1ar* expression may depend on changes in central ANG II synthesis and release. Not only is *Ace1* upregulated following CIH (14), but inhibition of ACE1 prevents CIH-mediated increases in MnPO FosB and hypertension (13). The increases in ANG II release observed in the current study may be dependent on changes in synthetic pathways and may contribute to the upregulation of *At1ar* expression. Furthermore, physiological stressors such as CIH may induce alternative ANG II signaling pathways in the MnPO that contribute to hypertension.

### Limitations

In a previous study, observations show that exogenously applied ANG II and ANG III increased calcium transients in ANG sniffer cells. In these studies, the sniffer cells were placed on brain slices containing the MnPO. Although previous studies demonstrated that the calcium transients were attenuated by inhibiting renin and ACE, it is possible that the AT1 receptors on the CHO cells were being activated by peptides structurally similar to ANG II instead of ANG II per se.

### Perspectives and Significance

Neuropeptides have different properties as compared with small molecule neurotransmitters (36, 37). They are typically synthesized as part of a larger protein that is enzymatically processed to release the neuropeptide and other possibly active peptides. After release, neuropeptide actions in the synapse are not terminated by reuptake but are instead enzymatically metabolized by extracellular enzymes into nonactive fragments. Alternatively, neuropeptides can diffuse out of the synapse and participate in volume transmission (36, 37). In the current study, we measured increases in the activity of ANG-sensitive sniffer cells placed on the MnPO that were associated with optogenetic activation and electrical stimulation of the SFO. Given the placement of the sniffer cells on the brain slice, TTX-dependent, extrasynaptic ANG II is likely to be responsible for their activation. This suggests that ANG II could participate in modulatory volume transmission. Our results also indicated that ANG II release is increased by CIH. It was previously shown that *At1ar*, *Ace1*, and *Ace2* are upregulated in the MnPO following CIH (26). Virally mediated knockdown of *At1ar* (25) and *Ace1* (13) in the MnPO prevents the development of the sustained increase in blood pressure associated with CIH. In male rats exposed to CIH, MnPO neurons have an ANG II-dependent reduction in GABA-mediated inhibition (38). Together these results indicate that CIH increases the synthesis and actions ANG II in the lamina terminalis in a way that supports increased blood pressure. If ANG II released from the lamina terminalis participates in volume transmission, its effects could extend to other parts of the neural axis.

## DATA AVAILABILITY

Data will be made available upon reasonable request.

## GRANTS

This work was supported by National Institutes of Health Grants P01 HL088052 and R01 HL155977 (to G.E.F. and J.T.C.).

## DISCLOSURES

No conflicts of interest, financial or otherwise, are declared by the authors.

## AUTHOR CONTRIBUTIONS

G.E.F. and J.T.C. conceived and designed research; G.E.F. performed experiments; G.E.F. analyzed data; G.E.F. and J.T.C. interpreted results of experiments; G.E.F. prepared figures; G.E.F. drafted manuscript; G.E.F. and J.T.C. edited and revised manuscript; G.E.F. and J.T.C. approved final version of manuscript.
